# Malignant Peritoneal Mesothelioma Presenting with High Protein, High Serum-Ascites Albumin Gradient

**DOI:** 10.7759/cureus.27286

**Published:** 2022-07-26

**Authors:** Zachary P Kerosky, Charleston R Powell, Phillip C Lindholm

**Affiliations:** 1 Internal Medicine, Madigan Army Medical Center, Tacoma, USA; 2 Internal Medicine, Weed Army Community Hospital, Fort Irwin, USA; 3 Gastroenterology, Madigan Army Medical Center, Tacoma, USA

**Keywords:** high serum ascites albumin gradient, dili, peritoneum, asbestos, saag, mesothelioma

## Abstract

Mesothelioma is a difficult-to-detect neoplasm that rarely develops in the peritoneum. In patients with unexplained ascites, pleural fluid analysis and ultrasonography is often the first step to achieving a diagnosis. This case report shares a unique presentation in which a patient who presented with unexplained ascites, was initially thought to have cirrhosis but was later found to have malignant peritoneal mesothelioma after cross-sectional imaging and tissue acquisition. This case illustrates the importance of a high clinical index of suspicion for mesothelioma given its variety of clinical presentations, as well as the utility of early cross-sectional imaging in such cases.

## Introduction

Mesothelioma is an insidious neoplasm that arises from serosal membranes of the pleura, pericardium, tunica vaginalis, and peritoneum. Of 3,300 cases diagnosed in the United States annually, 11% are peritoneal. Asbestos exposure is thought to be the predominant risk factor for the development of mesothelioma. The lifetime risk among those exposed to asbestos is as high as 10% with a 20-to-40-year latency from exposure to diagnosis [1]. Malignant peritoneal mesothelioma presents with abdominal pain, fatigue, increased abdominal girth, weight loss, and ascites. Given the non-specific nature of these symptoms, diagnosis is often delayed. Cross-sectional imaging, particularly computed tomography (CT) and positron emission tomography (PET) can aid in establishing a diagnosis, but confirmatory biopsy with a review of histology and immunohistochemical markers and molecular testing are needed to make a definitive diagnosis [2]. Treatment modalities include cytoreductive surgery, radiation, intraperitoneal chemotherapy, and systemic chemotherapy [3].

The serum-ascites albumin gradient (SAAG) is a diagnostic tool used to predict the presence or absence of portal hypertension. SAAG is calculated by subtracting the ascitic fluid albumin from the serum albumin. A value of <1.1 g/dL is thought to exclude portal hypertension and raises suspicion for etiologies like inflammation, infection, and malignancy. A value >1.1 g/dL predicts the presence of portal hypertension with 97% accuracy [2]. The SAAG may be elevated with any pathology that leads to portal hypertension. The differential for high SAAG ascites is subdivided into pre-hepatic, intra-hepatic, and post-hepatic.

Pre-hepatic etiologies include portal or splenic vein thrombosis or compression. Intra-hepatic etiologies include cirrhosis (alcoholic and otherwise), primary biliary cholangitis, primary sclerosis cholangitis, schistosomiasis, sarcoidosis, and more. Post-hepatic etiologies include constrictive pericarditis, congestive heart failure, and Budd-Chiari Syndrome. Total protein concentration is another tool to classify the ascitic fluid with a high SAAG. Concentrations greater than 2.5 g/dL are more suggestive of heart failure, and pericardial and veno-occlusive disease. Concentrations lower than this point more towards cirrhosis [4-5].

This case report shares a unique presentation in which a patient with high ascetic protein, high SAAG ascites was found to have peritoneal mesothelioma.

This article was previously presented as an e-poster at the American College of Gastroenterology (ACG) 2021 Annual Scientific Meeting & Postgraduate Course, Las Vegas, Nevada, United States, October 22-27, 2021. 

## Case presentation

A 69-year-old male with a history of myasthenia gravis who was diagnosed in 1979 and on methotrexate for approximately 20 years, rheumatic fever, and a history of testicular lymphoma in 1981 status-post left orchiectomy without chemotherapy or radiation, presented to his primary care physician with two months of abdominal swelling with early satiety. His family history includes unknown etiology of liver disease in his mother and sister from which the latter died from at age 52. He denied alcohol use and had no prior diagnosis of liver disease. Bedside ultrasonography showed large ascites and liver nodularity. His liver enzymes, international normalized ratio (INR), and albumin were normal. Formal ultrasound with Doppler showed a shrunken liver with large ascites concerning for decompensating cirrhosis. No evidence of thrombosis or venous outflow obstruction was found. The patient was referred to gastroenterology for new-onset ascites with concern for cirrhosis.

Methotrexate was stopped to prevent further hepatic injury. Ultrasound-guided paracentesis was performed and 8100 mL of serous fluid was obtained with SAAG of 1.3 and protein of 3.7 g/dL. Initial cytology did not reveal malignancy. Additional labs for Wilson’s disease, hemochromatosis, hepatitis A, B, C, and autoimmune hepatitis were negative. An echocardiogram revealed normal systolic function and no valvular pathology.

**Figure 1:**
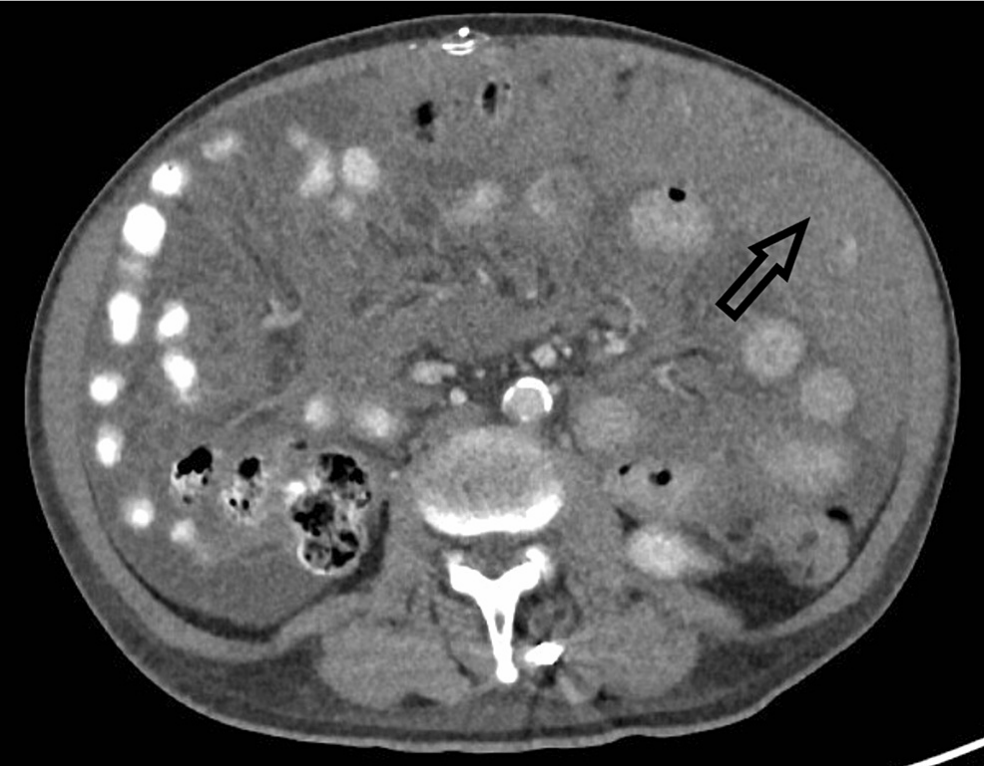
Contrasted abdominal CT showing diffuse omental thickening (indicated by arrow).

At this time, the etiology of the patient’s ascites remained unknown and plans were made for liver biopsy. Trans-hepatic, ultrasound-guided liver biopsy was obtained and showed overall mild and non-specific changes. There was no evidence of fibrosis, infiltrative disease, or venous outflow obstruction. Contrasted CT of chest, abdomen, and pelvis showed diffuse omental thickening concerning for peritoneal carcinomatosis (Figure 1).

**Figure 2:**
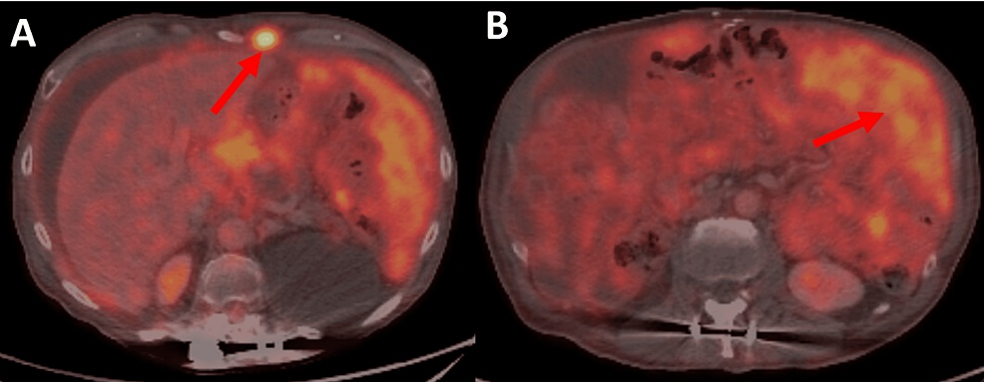
PET-CT fusion imaging with 2.4 x 1.5 cm hypermetabolic soft tissue nodule to left of xiphoid (indicated by arrow in image A) as well as markedly thickened omentum with moderate to marked metabolic activity (indicated by arrow in image B). PET-CT: positron emission tomography-computed tomography

A subsequent PET re-demonstrated these findings as well as showed a hypermetabolic 0.6 cm right upper lobe nodule, left hilar and bilateral anterior diaphragmatic lymph nodes, and a soft tissue nodule within the anterior chest wall to the left of the xiphoid all concerning for metastatic disease. No obvious primary tumor was identified, however. Plans were made to identify this through esophagogastroduodenoscopy (EGD), colonoscopy, and tissue acquisition of the chest wall nodule. The former two revealed no evidence of primary malignancy and the latter was biopsied and pathology was consistent morphologically and immunophenotypically with mesothelioma. The patient’s course continued with rapid accumulation of ascites requiring serial therapeutic paracenteses and ultimate placement of an indwelling peritoneal drain. He was referred to an outside institution oncology department after diagnosis of mesothelioma where he received palliative chemotherapy with carboplatin and pemetrexed (Figure 2).

## Discussion

Malignant peritoneal mesothelioma is an uncommon disease related to asbestos exposure that can present with peritoneal findings. Its indolent presentation often leads to delayed diagnosis due to non-specific symptoms and a lack of awareness about extra-pulmonary presentations.

This case began with a physical exam and ultrasound that pointed towards cirrhosis because of abdominal swelling and shrunken/nodular liver with large ascites on ultrasound. His family history of liver disease also raised concerns for development of non-alcoholic forms of cirrhosis when he denied alcohol use and prior diagnosis of liver disease. When these labs were negative, and extrahepatic thrombosis and venous outflow disorders were excluded, concern was primarily for other high SAAG forms of portal hypertension, like congestive heart failure. 

Because of normal systolic function ruling this out, a liver biopsy was obtained for what was still anticipated to be a problem with the liver itself. The biopsy ultimately yielded little and did not point towards liver pathology that would cause his presentation. Liver biopsy is the golden standard for diagnosing cirrhosis but has problems with sensitivity and specificity, itself. It is thought that reductions in sensitivity happen with biopsies less than 3 cm in length, fragmentation, and steatosis [5]. The patient's core biopsies were between 1.8 to 2.2 cm but there was no fragmentation or steatosis present. It is possible that significant fibrosis was missed but after finding a cause for the patient's ascites as well as a lack of evidence for portal hypertension on ultrasound and cross-sectional imaging, further biopsies were not pursued. The high SAAG and the negative cytology were certainly reasons for malignancy to be lower on the list of differential diagnoses initially. Several similar cases of high SAAG ascites ultimately determined to be malignant ascites were found including one with a fibrosarcoma that caused inferior vena cava (IVC) compression and resulting Budd-Chiari syndrome and another in which an ovarian tumor was implicated but no such metastases causing compression nor venous clots were found to explain a reason for portal hypertension [6-8]. It was postulated for the latter that fluid from the tumor itself may have been responsible for a falsely high SAAG. For our case, it is possible a similar disturbance occurred or that there was more advanced fibrosis missed by biopsy. 

The usual time from presenting symptoms to diagnosis of mesothelioma is four to six months and our patient required six months from symptom onset to tissue diagnosis. There are multiple studies demonstrating the sensitivity of a low SAAG for the exclusion of portal hypertension. As to its ability to help rule out malignancy, this is not well established. A small study comparing different ascitic fluid values between patients with malignant and non-malignant ascites shows the SAAG has a sensitivity of only 80% [9,10]. Cytology is thought to be even less sensitive for malignant ascites if the diagnosis is other than carcinomatosis. For example, sensitivity may be as low as 60%. This means that, in cases like this, early negative cytology should not remove malignancy from the differential alone. The discordance between the high SAAG and pathology led to delay in the diagnosis. Only after diagnosis was the patient found to have a significant occupational history of exposure to asbestos during his military career. 

## Conclusions

Peritoneal mesothelioma remains a rare and difficult diagnosis to make. When working up a patient with new ascites, there are limitations to peritoneal fluid analysis, cytology, and ultrasonography. We recommend broad, cross-sectional imaging early on in unexplained ascites as well as early discovery of occupational exposures. This case illustrates the importance of a high clinical index of suspicion for mesothelioma given its variety of clinical presentations.
